# Supplementary material for: Radial Access for Coronary Angiography Carries Fewer Complications Compared with Femoral Access: A Meta-Analysis of Randomized Controlled Trials
Source: J Clin Med. 2021 May 17;10(10):2163. doi: 10.3390/jcm10102163 (PMC8156941; doi:10.3390/jcm10102163)
Supplement: Supplementary file 1 [file jcm-10-02163-s001.zip › jcm-1175550-supplementary.pdf]

Supplementary Table S1. Main clinical, angiographic and procedural characteristics of the included studies.

Study	Year	TRA (nr)	TFA (nr)	Clinical Syndrome	Mean Age eyr.		Follow Up	Multicenter	Primary Endpoint	Primary Statistical Result	Sheatht size 5-6F/7F	Mace Definition
					TFA	TRA						
**ACCESS Kiemeneij et al [17]**	1997	300	300	Stable, ACS	61	62	30 days	No	1.Access site related endpoint 2.PTCA-related endpoints	1. p < 0.001 (radial better) 2. p = NS	100%/0%	Death, MI, Stroke CABG PTCA
**Achenbach et al [28]**	2008	152	155	Suspicious for Coronary artery disease	78	78	4 days	No	1.The success rate of TR coronary angiography and intervention Occurrence of major complications. 2. Minor complica-tions, procedural duration	1.p < 0.001 (radial better) 2. p < 0.001 (radial better)	100%/0%	Death Stroke Vascular access definition
**Akturk et al [41]**	2014	408	428	Suspicious for coronary artery disease	59.6	60.3	In Hospital	No	1.VAS scores after catheterisation	1.p < 0.0001 (Radial better)	-	Not defined
**Andrade et al [45]**	2017	120	120	NTSEMI Previous CAD	62.5	63.6	30 Days	No	1.Vascular complication at the arterial puncture site 30-days after the procedure.	N/S	-	Not defined
**Benit et al [18]**	1997	56	55	Symptomatic Ischemic heart disease	57.7	58.2	1 month	No	Entry site complications (bleeding, haematoma, transfusion, occlusion, surgery)	N/S	NR	Death Stent Thrombosis Repeat PTCA
**Bhat et al [46]**	2017	200	200	Primary PCI, Elective coronary Study and elective angioplasties.	61.8	60.6	In Hospital	No	1.Bleeding, access site, Non access site complications. Hospital stay duration Time of doing procedure	p = 0.0003 (radial better)	100%/0%	Not defined.
**Brueck et al [56]**	2009	512	512	Diagnostic or interventional Cardiac catheterization	63.2	64.1	In Hospital	No	Access Failure, procedural duration, Entry site complication, radiation exsposure.	p = 0.0008 (radial better)	85%/15%	Not defined
**Cooper et al [20]**	1999	101	99	Diagnostic Cardiac catheterization	60	60	1 week	No	Access site complication, stroke, median length of stay, bodily	p < 0.0001 (radial better)	100/0%	Not defined

									pain, back pain, walking ability			
**FARMI Brasselet et al [26]**	2007	57	57	STEMI, sustained chest pain	60	58	In Hospital	No	Peripheral arterial complications	p = 0.014 (radial better)	100%0%	Not defined
**FERARI Fastner et al [44]**	2017	200	200	Stable AP Unstable AP NSTEMI, STEMI, Pozitive viability testing, Controll angiography Syncope, HF.	69	71	30 Days	No	Access site bleeding, Re-hospitalization due to access site bleeding	(p < 0.05) (radial better)	N/A	Death MI Stenttrombosi s Stroke
**Gan et al [31]**	2009	95	105	STEMI	53.6	52.3	6 month	YES	N/A	N/A	-	Death, Re-Infarction, CABG Acces site complication,
**Hou et al [33]**	2010	100	100	STEMI	64.9	66.2	30 Days	No	MACE Vascular complication	N/S p < 0.01 (radial better)	-	-
**Mann et al [27]**	1998	68	77	STEMI.NSTEMI. ACS	63	62	In Hospital	No	Access site complication, Death, CABG, MI	N/A	-	Not defined
**MATRIX Valgimili et l [43]**	2015	4197	4207	STEMI, NSTEMI ACS	65.6	65.9	30 Days	YES	1.Composite of all-cause mortality, MI or stroke 2.Composite of major bleeding not related to CABG or MACE	p < 0.03 (radial better) p < 0.009 (radial better)	-	Death, MI StrokE
**OCEAN RACE Koltoswki et al [40]**	2014	52	51	STEMI	61	62.8	In Hospital	NO	Composite of major and minor bleeding	p = 0.238 N/S	100%	Stroke Death Major bleeding, Sudden Cardiac Arrest
**OCTOPLUS**	2004	192	185	Stable, NSTEMI,	82.6	83	In	YES	Composite vascular end npoint	p = 0.029	-	Not defined

**Louvard et [23]**				STEMI, ACS			Hospital		Comprising ≥of the following	(radial better)		
**RADIAL AMI Cantor et al [25]**	2005	25	58	STEMI	52	58	30 Days	YES	1.Reperfusion time<br>2.Major bleedin gaccess site complication	1.p = 0.04 (femoralbetter)<br>2.= NS	TRA:100%/0%<br>TFA:88%/12%	Not defined
**RADIAL CABG Michael et al [39]**	2013	64	64	STEMI	64	64	In Hospital	No	1.Volume of radiographic contrast administered During cardiac catheterization<br>2. MACE	1. p < 0.01) (femoralbetter)<br>2.N/S	-	MI Recurrent Angina Urgentrepeat TVR.
**RADIAMI Chodor et al [32]**	2009	50	50	STEMI	59.9	59.1	In Hospital	No	Not defined	N/A	100%/0%	Death, MI
**RADIAMI II Chodor et al [36]**	2011	49	59	STEMI	62.1	57.6	In Hospital	No	Not defined	N/A	100%/0%	Death, MI,CABG,TVR
**Reddy et al [22]**	2004	25	25	Diagnostic Cardiac catheterization	58	64	1 week	No	1.Sheath insertion time<br>2.Catherization time<br>3.Hemostazis time<br>4. Fluroscopy time<br>5.Contrast amount	P = N/S	-	Not defined
**RIFLE-STEACS Romangoli et al [37]**	2012	500	501	STEMI	65	65	30 Days	YES	NACE	P = 0.003 (radial better)	TRA:81.4%/18.6 %<br>TFA:90.8%/9.2%	Death, MI, Stroke. TLR
**RIVAL Jolly et al [35]**	2011	3507	3514	STEMI, NSTE ACS	62	62	30 Days	YES	Death, Myocardial Infarction, Stroke, non-CABG, related major bleeding.	P = 0.50	TRA:98.6%/1.4%<br>TFA:95.5%/4.5%	Death, MI, Stroke
**SAFARI-STEMI May et al [50]**	2020	1136	1156	STEMI	61.6	62	30 Days	YES	1.All cause mortality (bleeding)<br>2. Stroke, Reinfarction, Death	N/S	-	Not defined
**SAFE-PCI Koshy et al [48]**	2018	893	894	STABLE, PCI	65.2	64.3	72 Hours	YES	Post procedure bleeding or vascular complications	p = 0.3 (radial better)	-	Not defined
**Santas et al [429]**	2009	670	335	STABLE, STEMI	66	66	In Hospital	No	Percentage of procedure completed using the assigned aproach	p < 0.0001 (femoral better)	No	Not defined
**Scherthaner Et al [47]**	2018	125	125	STEMI, NSTE ACS	61.9	62.2	30 Days	YES	1.Occurrence hematoma<br>2.Local bleeding, the access site pseudoaneurism<br>3. Prolonged hospital stay	1.p < 0.0007 (radial better)<br>2.p = 0.04) (radial better)	TRA 8.8% / 91.2%//0.0% FA 0.8%/98.4%/0.8%	Not defined

**Slagboom Et al [24]**	2005	322	322	STABLE AP, UNSTABLE AP	60	60	1 month	No	1.Cardiac death<br>2.Emergency CABG<br>3. Myocardial Infarction<br>4.Major entry site complication	N/S	100%/0%	Cardiac death<br>Emergency CABG<br>MI<br>Major entry site complication
**STEMI RADIAL Bernat et al [42]**	2013	348	359	STEMI	62.7	61.5	30 Days	YES	1.Major bleeding and vascular access<br>site complication	p < 0.0001 (radial better)	TRA 100%/0%<br>TFA 92.2%/0.8%	Death<br>MI<br>Stroke
**SURF Nguen et al [59]**	2019	679	662	STEMI<br>NSTE ACS<br>STABLE AP<br>UNSTABLE AP	65	65	30 Days	No	1.ACUITY<br>2.Major Bleeding<br>3.MACE	(p = 0.013) (radial better)	NR	Death<br>Stroke<br>Myocardial Infarction<br>Urgent TLR
**TEMPURA Saito et al [21]**	2003	77	72	STEMI	66	67	In Hospital	No	MACE	p = 0.412) (similar)	NR	Death<br>MI<br>TLR
**Wang et al [38]**	2012	60	59	STEMI	59.8	60.2	In Hospital	No	1.Puncture time, cannulation time, and total procedure time.<br>2.Vascular access site complication, minor bleeding, major bleeding	p < 0.001 (Radial better)	NR	Death<br>Reinfarction<br>TVR
**Yan et al [27]**	2008	57	46	Acute Myocardial Infarction	70.3	71.4	30 Days	No	1.Vascular access site<br>2.MACE	1.P < 0.05 (radial better)<br>2.N/S	-	Death<br>Reinfarction<br>TVR
**Ziakas et al [34]**	2010	27	29	Clinical Indication for Coronary angiography	68.2	64.7	In hospital	No	1.MACE<br>2.Bleeding<br>3.Access site complication	1.N/S<br>2. N/S<br>3. P = 0.086 (radial better)	N/R	Death<br>Stroke<br>TVR

ACS = acutecoronarysyndrome; CABG = coronaryarterybypassgrafting; FARMI = FiveFrench Arterial Access With reopro inMyocardialInfarction; MACE = major adversecardiovascularevent(s); MATRIX =MinimizingAdverseHaemorrhagicEvents by Transradial Access Site andSystemicImplementationofAngiox; NA = not available; NACE = net adverse clinical event(s); NSTE = non–ST-segment elevation; OCEAN RACE = accessforpercutaneouscoronaryintervention in STEMI: radial vs. femoral-prospective; OUTCLAS = OutpatientCoronaryLow-Profile AngioplastyStudy; PTCA = percutaneoustransluminalcoronaryangioplasty; RADIAMI = Radial VersusFemoralApproachforPercutaneousCoronaryInterventions in

PatientsWithAcuteMyocardialInfarction; RIFLE-STEACS = Radial VersusFemoralRandomizedInvestigation in ST-ElevationAcuteCoronarySyndrome; RIVAL = Radial vsFemoral Access forCoronaryIntervention; SAFE-PCI = Studyof Access Site forEnhancementof PCI forWomen; STEMI = ST-segment elevationmyocardialinfarction; STEMI RADIAL = ST ElevationMyocardialInfarctiontreated by RADIAL or femoralapproach; TEMPURA = Test forMyocardialInfarction by ProspectiveUnicenterRandomizationfor Access Sites; TFA = transfemoralaccess; TRA = transradialaccess; AMI = Acute Myocardial Infarction; MACE = major adverse cardiac events; MI = myocardial infarction; NA = not applicable; No. = Numbers; NR = not reported; PCI = percutaneous coronary intervention; RADIAL AMI = Radial versusfemoralaccessforemergentpercutaneouscoronaryinterventionwithadjunctglycoproteinIIb/IIIainhibition in acutemyocardialinfarction; RADIAMI = Radial VersusFemoralApproachforPercutaneousCoronaryInterventions in PatientsWithAcuteMyocardialInfarction;TEMPURA = Test forMyocardialInfarction by ProspectiveUnicenterRandomizationfor Access Sites; TLR = targetlesionrevascularization; TVR = targetvesselrevascularization; MI:Myocardial Infarction

A) All-cause mortality

Study or Subgroup	Radial Events	Radial Total	Femoral Events	Femoral Total	Weight	Risk Ratio M-H, Random, 95% CI
Andrade 2017	0	120	0	120		Not estimable
FARMI 2007	3	57	3	57	1.5%	1.00 [0.21, 4.75]
Gan 2009	2	90	3	105	1.2%	0.78 [0.13, 4.55]
Hou 2010	4	100	5	100	2.2%	0.80 [0.22, 2.89]
Mann 1998	0	68	0	77		Not estimable
MATRIX 2015	66	4197	91	4207	36.8%	0.73 [0.53, 1.00]
OCEAN RACE 2014	1	52	3	51	0.7%	0.33 [0.04, 3.04]
RADIAL-AMI 2005	0	25	1	25	0.4%	0.33 [0.01, 7.81]
RADIAMI 2009	0	50	1	50	0.4%	0.33 [0.01, 7.99]
RADIAMI II 2011	0	49	0	59		Not estimable
RIFLE-STEACS 2012	26	500	46	501	16.8%	0.57 [0.36, 0.90]
RIVAL 2011	44	3507	51	3514	22.6%	0.86 [0.58, 1.29]
SAFARI-STEMI 2020	17	1136	15	1156	7.6%	1.15 [0.58, 2.30]
Scherthaner 2018	1	125	3	125	0.7%	0.33 [0.04, 3.16]
STEMI-RADIAL 2013	8	348	13	359	4.8%	0.63 [0.27, 1.51]
TEMPURA 2003	4	77	6	72	2.4%	0.62 [0.18, 2.12]
Wang 2012	0	60	1	59	0.4%	0.33 [0.01, 7.89]
Yan 2008	3	57	3	46	1.5%	0.81 [0.17, 3.81]
**Total (95% CI)**		**10618**		**10683**	**100.0%**	**0.74 [0.61, 0.89]**
Total events	179		245			

Heterogeneity: Tau² = 0.00; Chi² = 5.56, df = 14 (P = 0.98); I² = 0%
Test for overall effect: Z = 3.16 (P = 0.002)

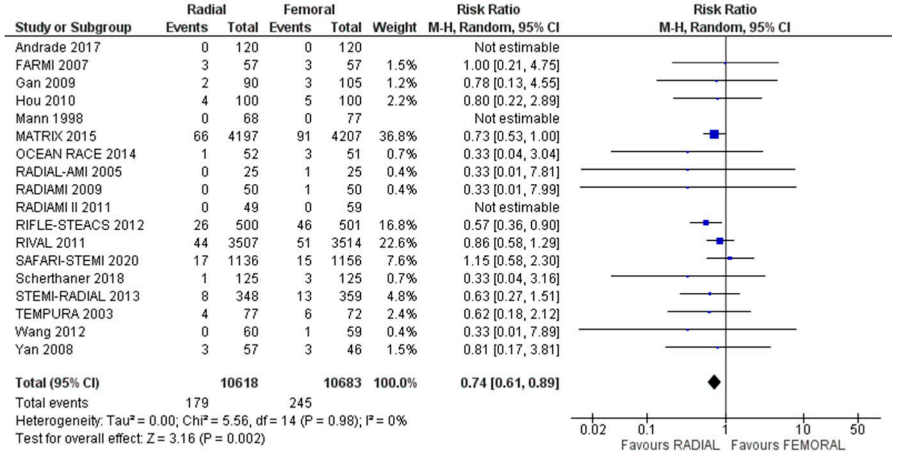

B) Major bleeding

Study or Subgroup	Radial Events	Radial Total	Femoral Events	Femoral Total	Weight	Risk Ratio M-H, Random, 95% CI
Andrade 2017	2	120	2	120	1.6%	1.00 [0.14, 6.98]
FARMI 2007	3	57	3	57	2.4%	1.00 [0.21, 4.75]
Gan 2009	0	90	2	105	0.7%	0.23 [0.01, 4.79]
Hou 2010	0	100	3	100	0.7%	0.14 [0.01, 2.73]
Mann 1998	0	0	0	0		Not estimable
MATRIX 2015	350	4197	606	4207	33.0%	0.58 [0.51, 0.66]
OCEAN RACE 2014	3	52	2	51	1.9%	1.47 [0.26, 8.44]
RADIAL-AMI 2005	0	25	0	25		Not estimable
RADIAMI 2009	3	50	7	50	3.3%	0.43 [0.12, 1.56]
RADIAMI II 2011	4	49	6	59	3.8%	0.80 [0.24, 2.68]
RIFLE-STEACS 2012	9	500	14	501	7.2%	0.64 [0.28, 1.47]
RIVAL 2011	183	3507	217	3514	29.6%	0.85 [0.70, 1.02]
SAFARI-STEMI 2020	10	1136	15	1156	7.6%	0.68 [0.31, 1.50]
Scherthaner 2018	0	125	1	125	0.6%	0.33 [0.01, 8.10]
STEMI-RADIAL 2013	5	348	26	359	5.8%	0.20 [0.08, 0.51]
TEMPURA 2003	0	772	72	0		Not estimable
Wang 2012	1	60	7	59	1.4%	0.14 [0.02, 1.11]
Yan 2008	0	57	1	46	0.6%	0.27 [0.01, 6.48]
**Total (95% CI)**		**11245**		**10534**	**100.0%**	**0.62 [0.48, 0.79]**
Total events	573		984			

Heterogeneity: Tau² = 0.04; Chi² = 22.39, df = 14 (P = 0.07); I² = 37%
Test for overall effect: Z = 3.78 (P = 0.0002)

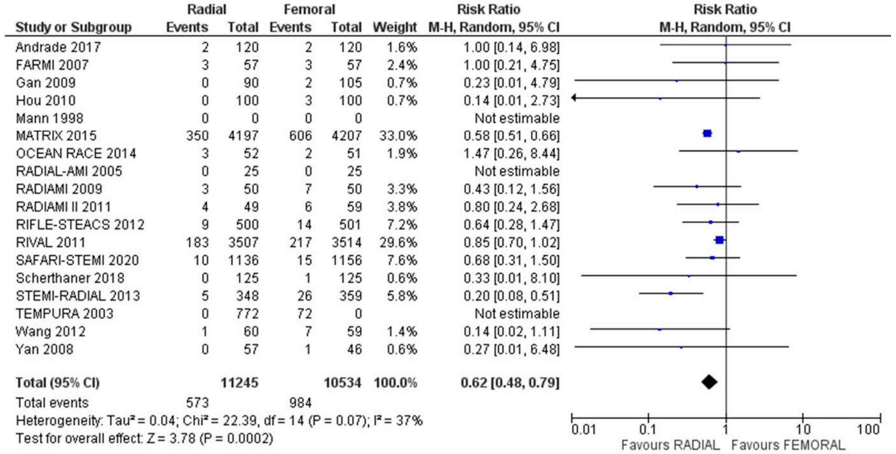

Supplementary Figure S1. Risk of all-cause mortality (**A**) and major bleeding (**B**) at follow-up: Radial vs. Femoral, in patients with acute coronary syndrome.

A) MACE

Study or Subgroup	Radial Events	Radial Total	Femoral Events	Femoral Total	Weight	Risk Ratio M-H, Random, 95% CI
Andrade 2017	3	120	5	120	0.6%	0.60 [0.15, 2.45]
FARMI 2007	7	57	7	57	1.2%	1.00 [0.37, 2.67]
Gan 2009	2	90	5	105	0.5%	0.47 [0.09, 2.35]
Hou 2010	4	100	5	100	0.7%	0.80 [0.22, 2.89]
Mann 1998	0	68	0	77		Not estimable
MATRIX 2015	381	4197	437	4207	69.8%	0.87 [0.77, 1.00]
OCEAN RACE 2014	5	52	6	51	0.9%	0.82 [0.27, 2.51]
RADIAL-AMI 2005	0	25	1	25	0.1%	0.33 [0.01, 7.81]
RADIAMI 2009	1	50	2	50	0.2%	0.50 [0.05, 5.34]
RADIAMI II 2011	1	49	1	59	0.2%	1.20 [0.08, 18.76]
RIFLE-STEACS 2012	36	500	57	501	7.5%	0.63 [0.42, 0.94]
RIVAL 2011	26	955	46	1003	5.3%	0.59 [0.37, 0.95]
SAFARI-STEMI 2020	45	1136	39	1156	6.7%	1.17 [0.77, 1.79]
Scherthaner 2018	1	125	6	125	0.3%	0.17 [0.02, 1.36]
STEMI-RADIAL 2013	12	348	15	359	2.1%	0.83 [0.39, 1.74]
TEMPURA 2003	13	77	16	72	2.8%	0.76 [0.39, 1.47]
Wang 2012	2	60	6	59	0.5%	0.33 [0.07, 1.56]
Yan 2008	3	57	3	46	0.5%	0.81 [0.17, 3.81]
**Total (95% CI)**		**8066**		**8172**	**100.0%**	**0.83 [0.75, 0.93]**
Total events	542		657			

Heterogeneity: Tau² = 0.00; Chi² = 12.00, df = 16 (P = 0.74); I² = 0%
Test for overall effect: Z = 3.25 (P = 0.001)

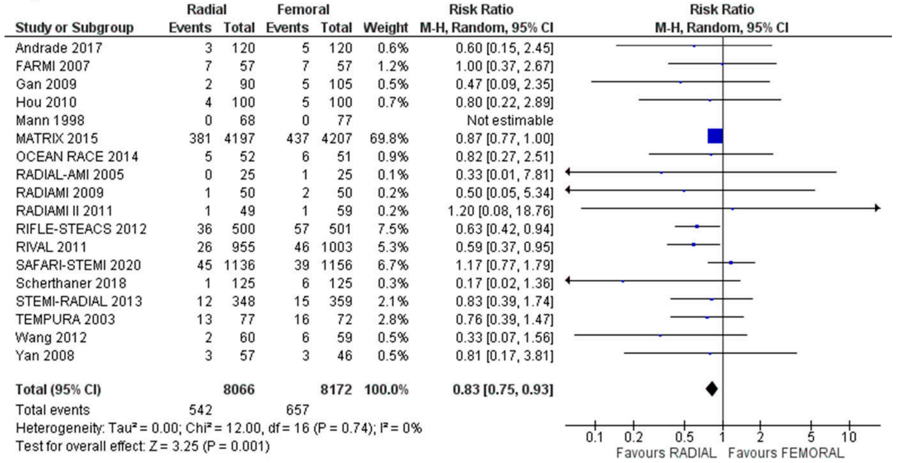

B) Major vascular complications

Study or Subgroup	Radial Events	Radial Total	Femoral Events	Femoral Total	Weight	Risk Ratio M-H, Random, 95% CI
Andrade 2017	9	120	15	120	9.5%	0.60 [0.27, 1.32]
FARMI 2007	2	57	11	57	3.6%	0.18 [0.04, 0.78]
Gan 2009	2	90	12	105	3.5%	0.19 [0.04, 0.85]
Hou 2010	0	100	5	100	1.0%	0.09 [0.01, 1.62]
Mann 1998	0	68	3	77	1.0%	0.16 [0.01, 3.07]
MATRIX 2015	4	4197	15	4204	5.7%	0.27 [0.09, 0.80]
OCEAN RACE 2014	0	0	0	0		Not estimable
RADIAL-AMI 2005	1	25	1	25	1.1%	1.00 [0.07, 15.12]
RADIAMI 2009	5	50	8	50	6.2%	0.63 [0.22, 1.78]
RADIAMI II 2011	8	49	12	59	9.1%	0.80 [0.36, 1.81]
RIFLE-STEACS 2012	1	500	2	501	1.4%	0.50 [0.05, 5.51]
RIVAL 2011	49	3507	131	3514	21.7%	0.37 [0.27, 0.52]
SAFARI-STEMI 2020	37	1136	46	1156	18.3%	0.82 [0.54, 1.25]
Scherthaner 2018	11	125	31	125	12.3%	0.35 [0.19, 0.67]
STEMI-RADIAL 2013	1	348	3	359	1.6%	0.34 [0.04, 3.29]
TEMPURA 2003	0	0	0	0		Not estimable
Wang 2012	1	60	7	59	1.9%	0.14 [0.02, 1.11]
Yan 2008	1	57	6	46	1.9%	0.13 [0.02, 1.08]
**Total (95% CI)**		**10489**		**10557**	**100.0%**	**0.44 [0.33, 0.59]**
Total events	132		308			

Heterogeneity: Tau² = 0.08; Chi² = 20.57, df = 15 (P = 0.15); I² = 27%
Test for overall effect: Z = 5.42 (P < 0.00001)

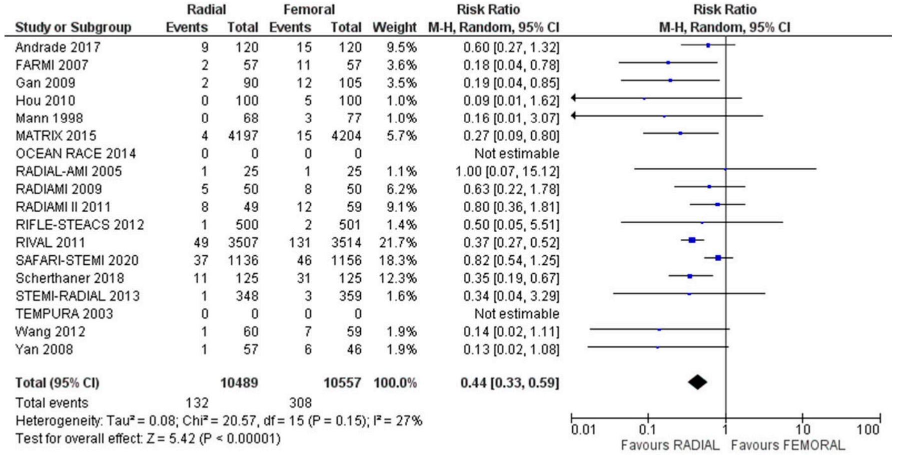

C) Myocardial infarction

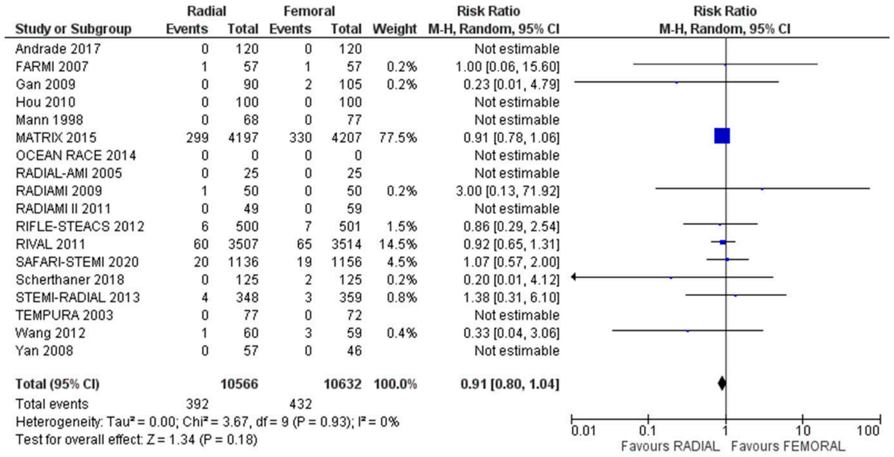

D) Stroke

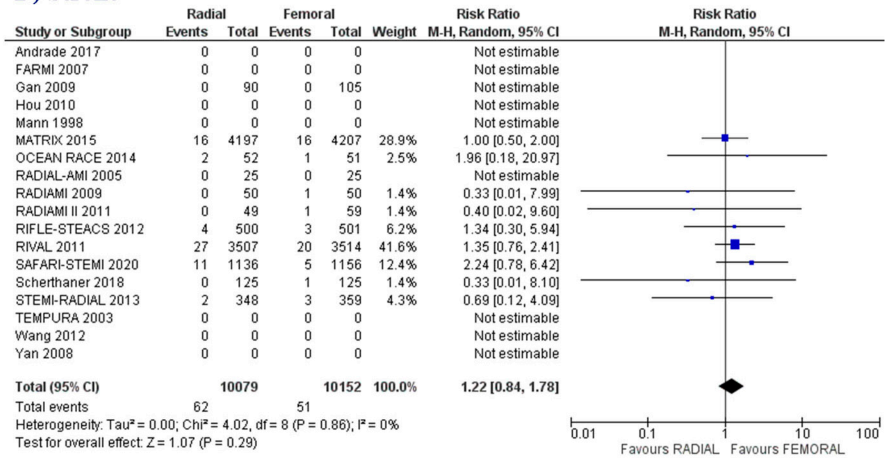

Supplementary Figure S2. Risk of MACE (**A**) and major vascular complications (**B**) at follow-up: Radial vs. Femoral, in patients with acute coronary syndrome. Risk of myocardial infarction (**C**) and stroke (**D**) at follow-up: Radial vs. Femoral, in patients with acute coronary syndrome.

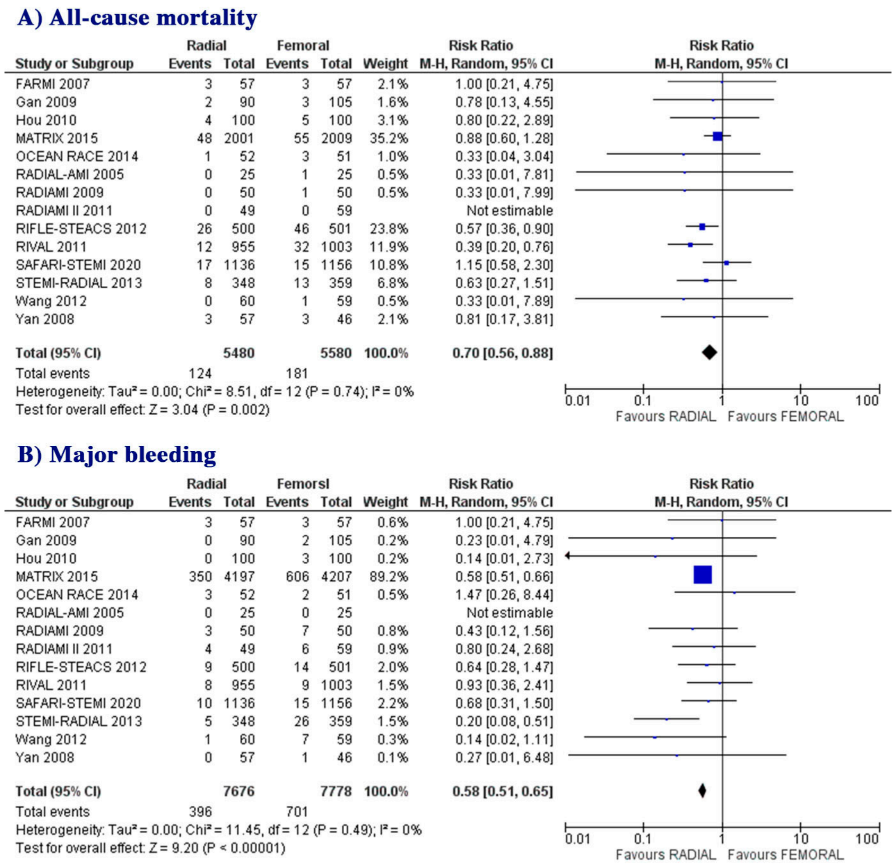

Supplementary Figure S3. Risk of all causes mortality (**A**) and major bleeding (**B**) at follow-up: Radial vs. Femoral, in patients with STEMI.

A) MACE

Study or Subgroup	Radial Events	Radial Total	Femoral Events	Femoral Total	Weight	Risk Ratio M-H, Random, 95% CI
FARMI 2007	7	57	7	57	2.6%	1.00 [0.37, 2.67]
Gan 2009	2	90	5	105	1.0%	0.47 [0.09, 2.35]
Hou 2010	4	100	5	100	1.5%	0.80 [0.22, 2.89]
MATRIX 2015	121	2001	126	2009	43.5%	0.96 [0.76, 1.23]
OCEAN RACE 2014	5	52	6	51	2.0%	0.82 [0.27, 2.51]
RADIAL-AMI 2005	0	25	1	25	0.3%	0.33 [0.01, 7.81]
RADIAMI 2009	1	50	2	50	0.5%	0.50 [0.05, 5.34]
RADIAMI II 2011	1	49	1	59	0.3%	1.20 [0.08, 18.76]
RIFLE-STEACS 2012	36	500	57	501	16.0%	0.63 [0.42, 0.94]
RIVAL 2011	26	955	46	1003	11.4%	0.59 [0.37, 0.95]
SAFARI-STEMI 2020	45	1136	39	1156	14.3%	1.17 [0.77, 1.79]
STEMI-RADIAL 2013	12	348	15	359	4.6%	0.83 [0.39, 1.74]
Wang 2012	2	60	6	59	1.0%	0.33 [0.07, 1.56]
Yan 2008	3	57	3	46	1.1%	0.81 [0.17, 3.81]
**Total (95% CI)**		**5480**		**5580**	**100.0%**	**0.85 [0.72, 0.99]**
Total events	265		319			

Heterogeneity: $Tau^2 = 0.00$; $Chi^2 = 10.31$, df = 13 (P = 0.67); $I^2 = 0\%$
Test for overall effect: Z = 2.07 (P = 0.04)

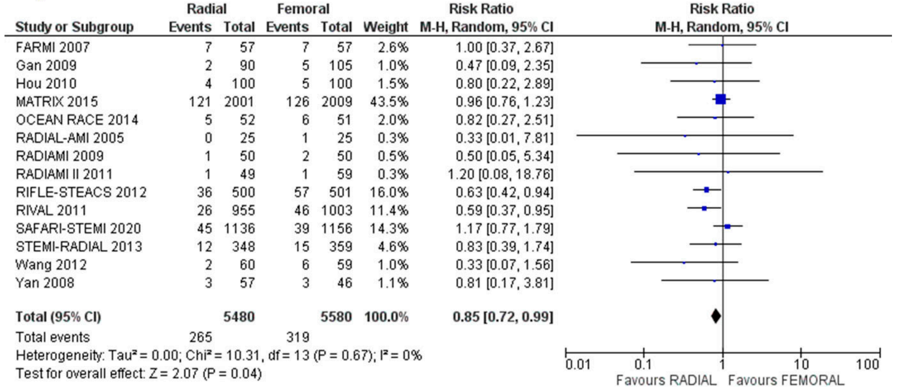

B) Major vascular complications

Study or Subgroup	Femoral Events	Femoral Total	Radial Events	Radial Total	Weight	Risk Ratio M-H, Random, 95% CI
FARMI 2007	2	57	11	57	6.0%	0.18 [0.04, 0.78]
Gan 2009	2	90	12	105	6.0%	0.19 [0.04, 0.85]
Hou 2010	0	100	5	100	1.8%	0.09 [0.01, 1.62]
MATRIX 2015	4	4197	15	4204	9.2%	0.27 [0.09, 0.80]
OCEAN RACE 2014	0	0	0	0		Not estimable
RADIAL-AMI 2005	1	25	1	25	2.0%	1.00 [0.07, 15.12]
RADIAMI 2009	5	50	8	50	9.9%	0.63 [0.22, 1.78]
RADIAMI II 2011	8	49	12	59	13.5%	0.80 [0.36, 1.81]
RIFLE-STEACS 2012	1	500	2	501	2.6%	0.50 [0.05, 5.51]
RIVAL 2011	12	955	35	1003	16.9%	0.36 [0.19, 0.69]
SAFARI-STEMI 2020	37	1136	46	1156	22.7%	0.82 [0.54, 1.25]
STEMI-RADIAL 2013	1	348	3	359	2.8%	0.34 [0.04, 3.29]
Wang 2012	1	60	7	59	3.3%	0.14 [0.02, 1.11]
Yan 2008	1	57	6	46	3.3%	0.13 [0.02, 1.08]
**Total (95% CI)**		**7624**		**7724**	**100.0%**	**0.43 [0.29, 0.64]**
Total events	75		163			

Heterogeneity: $Tau^2 = 0.14$; $Chi^2 = 17.31$, df = 12 (P = 0.14); $I^2 = 31\%$
Test for overall effect: Z = 4.11 (P < 0.0001)

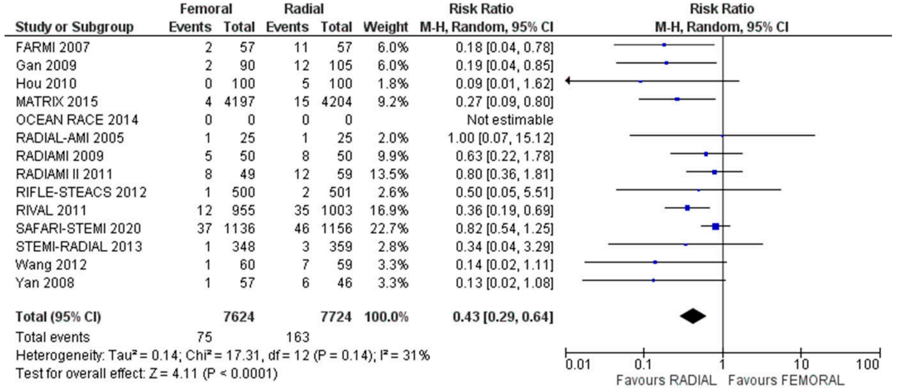

C) Myocardial infarction

Study or Subgroup	Radial Events	Radial Total	Femoral Events	Femoral Total	Weight	Risk Ratio M-H, Random, 95% CI
FARMI 2007	1	57	1	57	0.2%	1.00 [0.06, 15.60]
Gan 2009	0	90	2	105	0.2%	0.23 [0.01, 4.79]
Hou 2010	0	100	0	100		Not estimable
MATRIX 2015	299	4197	330	4207	77.7%	0.91 [0.78, 1.06]
OCEAN RACE 2014	0	0	0	0		Not estimable
RADIAL-AMI 2005	0	25	0	25		Not estimable
RADIAMI 2009	1	50	0	50	0.2%	3.00 [0.13, 71.92]
RADIAMI II 2011	0	49	0	59		Not estimable
RIFLE-STEACS 2012	6	500	7	501	1.5%	0.86 [0.29, 2.54]
RIVAL 2011	60	3507	65	3514	14.6%	0.92 [0.65, 1.31]
SAFARI-STEMI 2020	20	1136	19	1156	4.5%	1.07 [0.57, 2.00]
STEMI-RADIAL 2013	4	348	3	359	0.8%	1.38 [0.31, 6.10]
Wang 2012	1	60	3	59	0.4%	0.33 [0.04, 3.06]
Yan 2008	0	57	0	46		Not estimable
**Total (95% CI)**		**10176**		**10238**	**100.0%**	**0.92 [0.80, 1.05]**
Total events	392		430			

Heterogeneity: Tau² = 0.00; Chi² = 2.70, df = 8 (P = 0.95); I² = 0%
Test for overall effect: Z = 1.29 (P = 0.20)

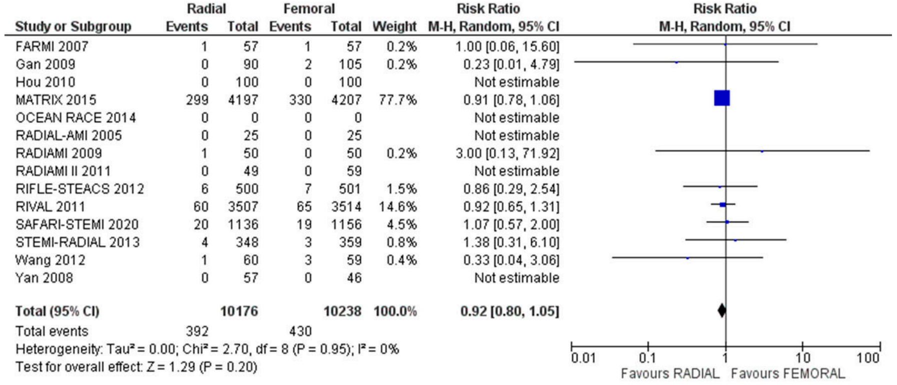

D) Stroke

Study or Subgroup	Radial Events	Radial Total	Femoral Events	Femoral Total	Weight	Risk Ratio M-H, Random, 95% CI
FARMI 2007	0	0	0	0		Not estimable
Gan 2009	0	90	0	105		Not estimable
Hou 2010	0	0	0	0		Not estimable
MATRIX 2015	16	4197	16	4207	29.3%	1.00 [0.50, 2.00]
OCEAN RACE 2014	2	52	1	51	2.5%	1.96 [0.18, 20.97]
RADIAL-AMI 2005	0	25	0	25		Not estimable
RADIAMI 2009	0	50	1	50	1.4%	0.33 [0.01, 7.99]
RADIAMI II 2011	0	49	1	59	1.4%	0.40 [0.02, 9.60]
RIFLE-STEACS 2012	4	500	3	501	6.3%	1.34 [0.30, 5.94]
RIVAL 2011	27	3507	20	3514	42.2%	1.35 [0.76, 2.41]
SAFARI-STEMI 2020	11	1136	5	1156	12.6%	2.24 [0.78, 6.42]
STEMI-RADIAL 2013	2	348	3	359	4.4%	0.69 [0.12, 4.09]
Wang 2012	0	0	0	0		Not estimable
Yan 2008	0	0	0	0		Not estimable
**Total (95% CI)**		**9954**		**10027**	**100.0%**	**1.25 [0.86, 1.81]**
Total events	62		50			

Heterogeneity: Tau² = 0.00; Chi² = 3.37, df = 7 (P = 0.85); I² = 0%
Test for overall effect: Z = 1.15 (P = 0.25)

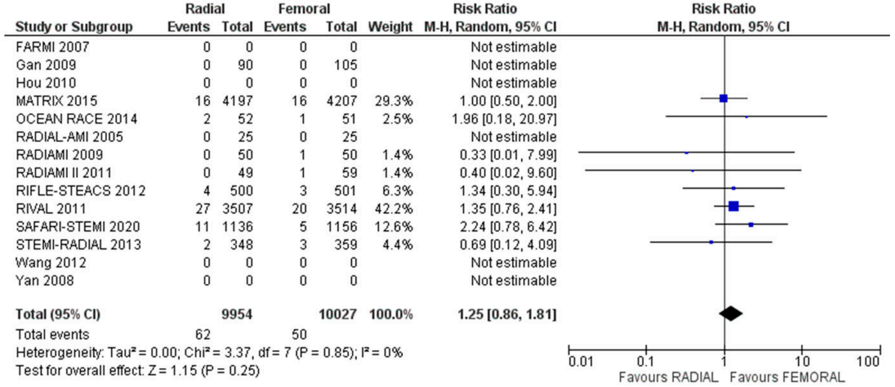

Supplementary Figure S4. Risk of MACE (**A**) and major vascular complications (**B**) at follow-up: Radial vs. Femoral, in patients with STEMI. Risk of myocardial infarction (**C**) and stroke (**D**) at follow-up: Radial vs. Femoral, in patients with STEMI.

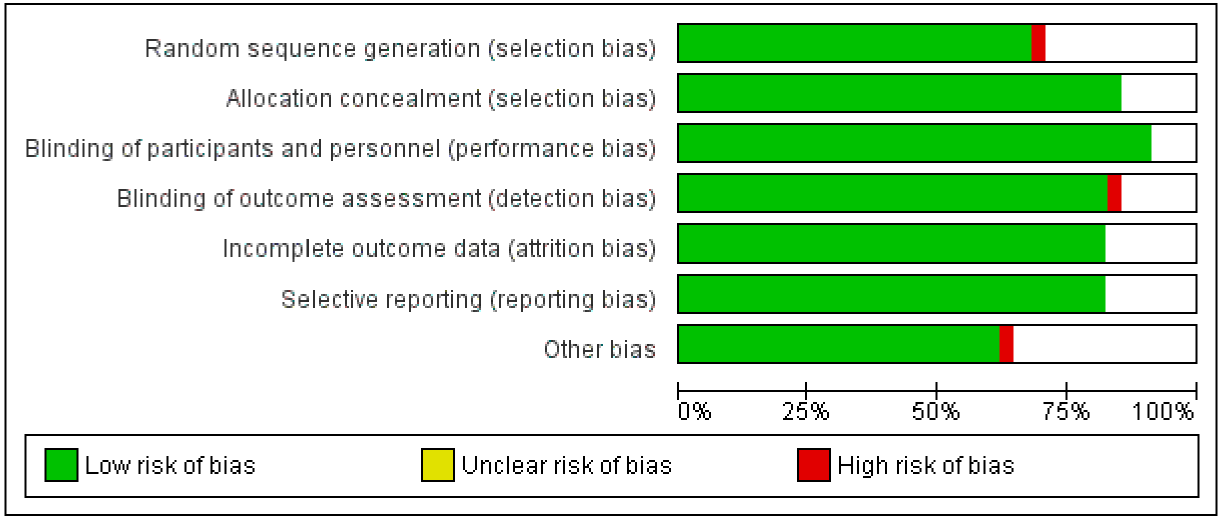

Supplementary Figure S5. Risk of bias pf the included studies.
